# Effect of percutaneous and arthroscopically assisted osteosynthesis of talar body fractures

**DOI:** 10.1186/s12891-022-05991-6

**Published:** 2022-12-14

**Authors:** Yong Hu, Zhengxun Li, Yang Wang, Ning Zhang, Wenpeng Xu, Xiucun Li

**Affiliations:** Department of Hand and Foot Surgery, The Second Hospital of Shandong University, No.247, Beiyuan Street, 250033 Jinan, Shandong People’s Republic of China

**Keywords:** Talar body fractures, Arthroscopy, Percutaneous osteosynthesis

## Abstract

**Background:**

Talar fractures are relatively uncommon, and the complex anatomy of the talus impedes their visualization, reduction, and fixation without performing an arthrotomy or osteotomy. To date, few studies have evaluated the complications of arthroscopically assisted percutaneous talar osteosynthesis. This clinical retrospective study aimed to investigate the effectiveness of this procedure according to the complications and functional outcomes.

**Methods:**

Arthroscopically assisted percutaneous talar osteosynthesis was performed in 15 patients (10 men and 5 women) with 16 fractures (one bilateral). The mean patient age was 31 years (range, 14–52 years). The Sneppen classification of the fractures was type II in 14 cases and type III in 2 cases.

**Results:**

Fifteen patients were followed up for 36 months on average (range, 18–65 months). No skin infection, osteomyelitis, or skin necrosis was observed in any patient. During the follow-up, no bony non-union or delayed union was found. At the final follow-up, 2 out of the 15 patients (13.3%) had peri-talar osteoarthritis. The ankle–hindfoot pain was absent in 11 patients (12 ankles) and mild in 4 patients. Based on the American Orthopaedic Foot and Ankle Society (AOFAS) ankle–hindfoot scale, functional results were excellent in 7 ankles and good in 9 ankles. The mean AOFAS ankle–hindoot score of the patients was 85.7 (range, 79–93).

**Conclusion:**

Arthroscopically assisted percutaneous talar osteosynthesis is a reliable and feasible technique that yields good clinical outcomes.

## Background

Talar fractures are usually caused by high-energy traumas, such as motor-vehicle accidents and high-fall injuries. Talar fractures are relatively uncommon, with an estimated incidence of 0.1–0.85% [1], and account for 3–5% of foot-and-ankle fractures [2]. The talus has a unique anatomical structure, in which over half of the surface is covered by the articular cartilage and there are no muscular attachments [3]. Furthermore, the complicated anatomy of the talus impedes the visualization, reduction, and fixation of fractures [4]. Thus, the treatment of talar fractures poses a serious challenge to orthopedic surgeons.

Based on their anatomic regions, talar fractures are divided into the fractures of the head, neck, and body [2], of which talar body fractures account for 7–38% of all talar fractures [5]. Talar body fractures are one of the most difficult fractures to operatively treat in foot-and-ankle surgery [6]. According to the pattern and location, Sneppen et al. have classified talar body fractures into the following five types: (I) compression or osteochondral dome fracture; (II) coronal, sagittal, or horizontal shear fracture; (III) posterior tubercle fracture; (IV) lateral tubercle fracture; and (V) crushed/comminuted fracture [7]. Currently, conventional open reduction-internal fixation (ORIF) is often recommended to treat displaced talar body fractures [5, 8], and various surgical approaches have been used to operatively reduce and fix talar body fractures [8]. If necessary, a medial or lateral malleolar osteotomy is used as an alternative or auxiliary method to the anteromedial or anterolateral approach to the talus [6, 8]. Unfortunately, these surgical techniques can cause high rates of postoperative complications and poor prognosis. Vallier et al. have reported that the talar body fractures treated with ORIF have a 38.5% rate of osteonecrosis, a 65.4% rate of arthritis in the ankle joint, and a 34.6% rate of arthritis in the sub-talar joint [5]. Biz et al. have concluded that the complications of talar body fractures treated with ORIF include malunions (21.4%), wound problems (25%), avascular necrosis (25%), and post-traumatic arthritis (78.6%) [9].

In 2012, Abdelgaid et al. reported the percutaneous reduction and screw fixation for the treatment of talar neck fractures [10], and in 2014, Jorgensen et al. reported the surgical techniques for arthroscopic treatment of talar body fractures [11]. To date, few studies have evaluated the complications of arthroscopically assisted percutaneous osteosynthesis of talar body fractures. Importantly, the effectiveness of this procedure in displaced talar body fractures is yet to be determined. Thus, this clinical retrospective study aimed to investigate the effectiveness of arthroscopically assisted percutaneous osteosynthesis in displaced talar body fractures.

## Methods

**Fig. 1:**
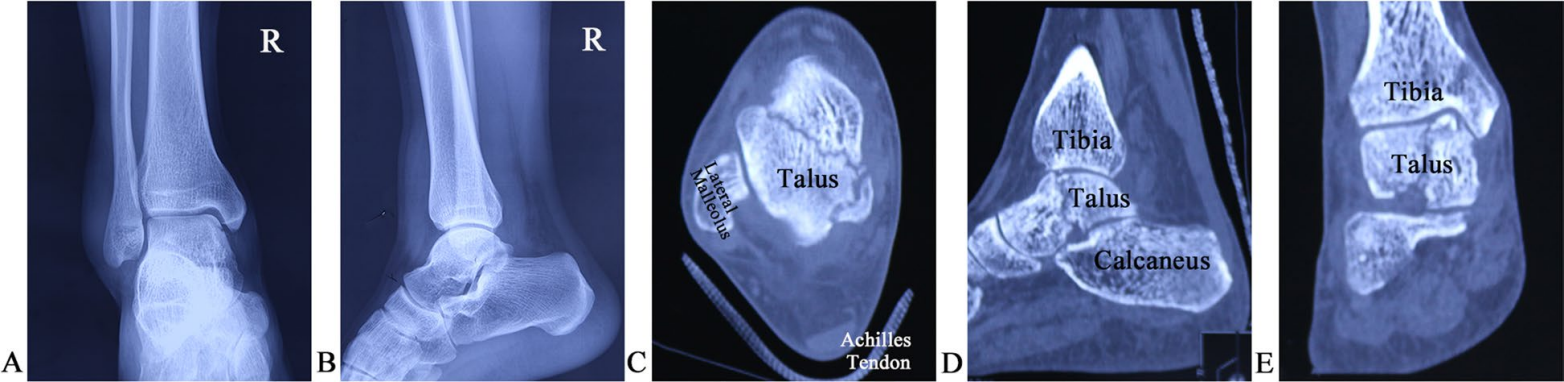
Preoperative anteroposterior (**A**) and lateral (**B**) X-ray radiographs, and axial (**C**), sagittal (**D**), and coronal (**E**) computed tomography scans of a 30-year-old male patient with a right talar body fracture (Sneppen II)

This study was approved by the research ethics committee of the Second Hospital of Shandong University. All the methods were carried out in accordance with the WMA Declaration of Helsinki ethical principles for medical research involving human subjects. Patients who suffered from displaced talar body fractures of Sneppen type I–IV [7] with articular displacement > 2 mm were selected for the study (Fig. 1). Furthermore, patients with multiple (ankle, sub-talar, or talonavicular) joint dislocations permissive to closed reduction, or bilateral fractures were also included in the study. Patients with displaced talar body fractures with articular displacement ≤ 2 mm or fractures of Sneppen type V were excluded. Accordingly, we reviewed the medical records of inpatients and found that 17 patients with talar body fractures underwent arthroscopically assisted percutaneous osteosynthesis between June 2016 and October 2019. Of these patients, 2 were excluded during the follow-up period because they discontinued this study. Finally, 15 patients were included in the study analyses.

### Surgical technique

All the patients underwent operative treatment under general anesthesia. The tourniquet was used on the thigh of the affected side. Before the surgery, X-ray radiography and computed tomography (CT) (Fig. 1) were used to assess the anatomical location and severity of each fracture, and the condition of joint dislocation, for the choice of the surgical approach. When the talar body fracture was combined with an ankle, sub-talar, or talonavicular joint dislocation, the reduction of the joint dislocation was performed immediately after the trauma.

**Fig. 2:**
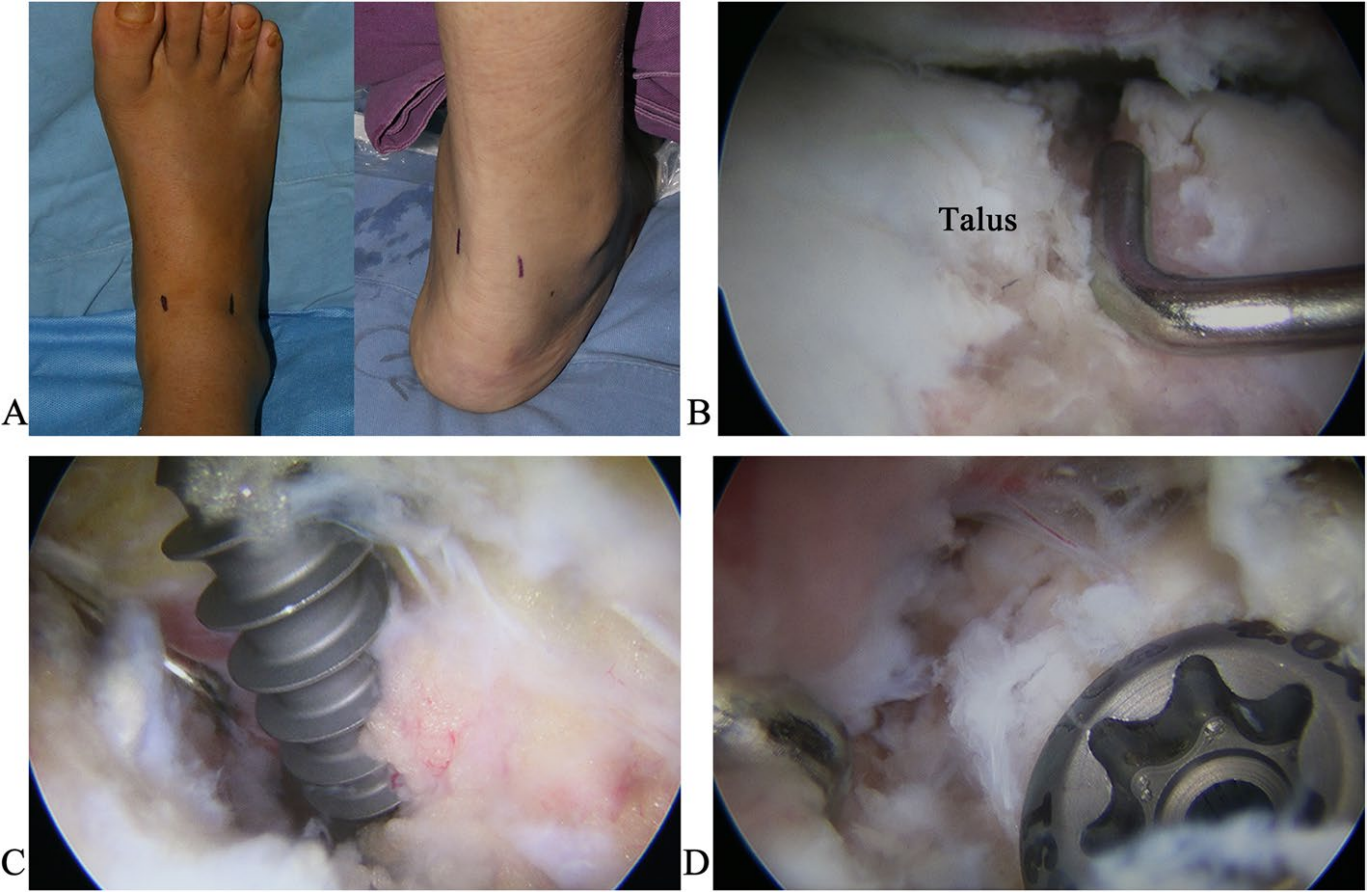
The procedure of arthroscopically assisted percutaneous talar osteosynthesis. **A** The anteromedial and anterolateral arthroscopy portals (left), and the posteromedial and posterolateral arthroscopy portals (right). **B** The fracture fragments were raised and reduced using a straight microprobe and osteotome. **C** 4.0-mm-diameter hollow screws were embedded. **D** The reduction state of the fractures was assessed

**Fig. 3:**
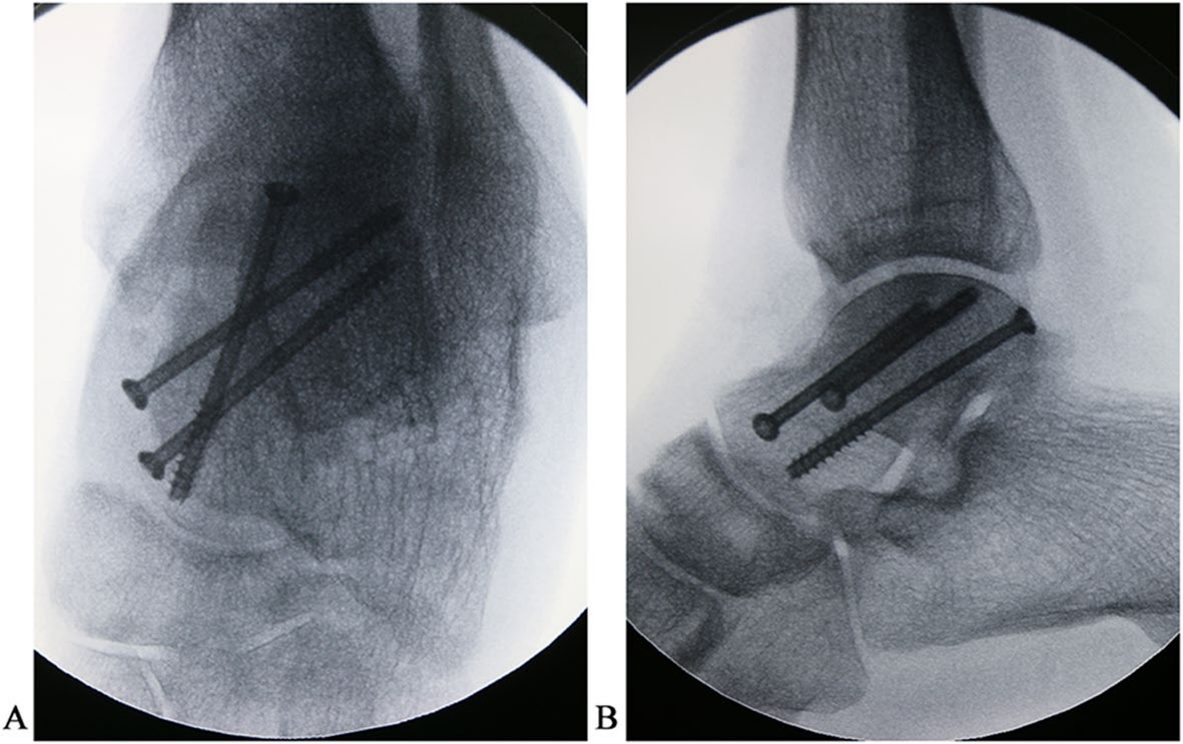
Immediate postoperative anteroposterior (**A**) and lateral (**B**) X-ray radiographs of the talar body fractures treated with arthroscopically assisted percutaneous osteosynthesis

If the fracture was in the front two-thirds of the talar body, the patient was initially placed in the supine position. The anteromedial and anterolateral ankle arthroscopy portals were used. The anteromedial portal is located medially at the junction between the anterior line of the ankle joint and the tibialis anterior tendon. The anterolateral portal is situated lateral to the junction between the anterior line of the ankle joint and the peroneus tertius tendon. A 4.0-mm-diameter arthroscope with a 30^°^ oblique viewing angle was used to visualize the condition of the fracture and articular surface displacement. Under the arthroscopic visualization, the hematoma, minuscule bone fragments, and inflammatory tissue were removed using a 4.0-mm-diameter cutter, with the aid of a straight microprobe and straight linvatec grasping forceps. The fracture fragments were raised and reduced using the straight microprobe and osteotome under arthroscopic visualization. A 2.0-mm-diameter Kirschner wire was embedded under arthroscopic visualization to temporarily stabilize the fracture. If there was a non-constant bony fragment, it was fixed using a 2.5-mm-diameter hollow screw. Afterward, the patient was positioned in the prone position. The posterolateral and posteromedial ankle arthroscopy portals, adjacent to the Achilles tendon, were used. The posteromedial portal was placed lateral to the flexor hallucis longus tendon to protect the neurovascular bundle from injury. Under arthroscopic visualization, the hematoma, minuscule bone fragments, and inflammatory tissue behind the ankle joint were resected using a 4.0-mm-diameter cutter. The flexor hallucis longus tendon was exposed and then retracted medially. The extent of damage to the ankle and sub-talar joints were assessed through the arthroscopic posterior approach while the fracture blocks were raised and reduced and then temporarily fixed using Kirschner wires. The reduction states of the talar body fractures at the ankle and sub-talar joints were assessed via the arthroscopic posterior approach. Intraoperative x-ray radiography was used to ensure that the guide pin was located in the middle of the talar head. Afterward, 4.0 mm-diameter hollow screws were inserted, and all the Kirschner wires were removed (Figs. 2 and 3).

### Postoperative management

The compression dressing of the limb on the affected side was performed within one week of the operation. The skin sutures were removed two weeks after the operation, and a short-leg non-walking cast was post-operatively worn for 8 weeks. Functional exercises of the ankle and sub-talar joints were performed. Partial weight-bearing walking on the affected side was allowed 9–10 weeks after the operation, and full weight-bearing walking was started on week 12.

### Functional evaluation

The quality of the reduction of each talar body fracture was evaluated via intraoperative fluoroscopy and post-operative CT. At the final follow-up, a radiographic examination of the anteroposterior and lateral views of the ankle was performed to assess malunion, nonunion, peri-talar osteoarthritis, and osteonecrosis. Malunion and nonunion were defined as non-anatomic healing of the talus and lack of bony healing within one year, respectively [12]. Peri-talar osteoarthritis was defined as periarticular osteophyte formation, narrowing of the joint space, or development of sub-chondral sclerosis or cysts, but was not graded according to its severity [13]. Based on the X-ray radiographs, any area with a denser talar dome than the adjacent structures was considered to have osteonecrosis (Hawkins sign) [13]. The pain level at the limb on the affected -side was evaluated using the visual analog scale (VAS), ranging between 0 and 10 points (0, no pain; 1–3, mild pain; 4–6, moderate pain; and 7–10, severe pain) [14]. Wound scar was measured using the modified Vancouver scar scale (mVSS), ranging from 0 to 15 points (pigmentation, 0–3; vascularity, 0–3; pliability, 0–5; and height: 0–4), in which the severity of the scar increases with the score [15]. Clinical outcomes were assessed based on the American Orthopaedic Foot and Ankle Society (AOFAS) ankle–hindfoot scale [16], and the Manchester-Oxford foot questionnaire (MOxFQ) [17, 18]. According to the guidelines of the AOFAS [16], the range of motion (ROM) of the ankle joint was evaluated with the ankle in the neutral position as follows: normal or mild restriction (≥ 30^o^) was assessed as three points, moderate restriction (15–29^o^) was assessed as two points, and severe restriction (< 15^o^) was assessed as one point. The ROM of the sub-talar joint was also assessed with the ankle in the neutral position as follows: normal or mild restriction (75–100% normal) was evaluated as three points, moderate restriction (25–74% normal) was evaluated as two points, and severe restriction (< 25% normal) was evaluated as one point. Finally, the AOFAS ankle–hind-foot score was grouped into excellent (90–100 points), good (75–89 points), fair (50–47 points), and poor (< 50 points) [16, 19].

MOxFQ is a specific patient-reported outcome measure of foot and ankle injuries and comprises the following three parts: foot pain, walking/standing problems, and issues related to social interaction, including feelings of self-consciousness about foot/footwear appearance. The corresponding scale ranges from 0 to 100 points, with 0 points representing an optimal result [17, 18].

## Results

### Baseline information

The average follow-up period of the 15 patients (10 men and 5 women) was 36 months (range, 18–65 months). The mean age of the patients at the time of surgery was 31 years (range, 14–52 years). None of the patients had diabetes mellitus, hypertensive disease, or soft-tissue swelling with fracture blisters. One patient had bilateral talar body fractures, whereas the remaining patients had unilateral fractures, of which 5 were on the left, and 11 were on the right. Of the 16 fractures in total, 14 and 2 were Sneppen type II and III fractures, respectively. Additionally, 3 out of the 15 patients had a talar body fracture with joint dislocation (ankle and sub-talar joint = 1, sub-talar and talonavicular joint = 2). The most common cause of injury was high falls (*n* = 9). The basic information of the 15 patients is shown in Table 1.

**Table 1:** Basic information of the 15 patients with 16 talar body fractures

Case	Gender	Laterality	Injury Mechanism	Sneppen Type	Joint Dislocation	Initial Reduction	Complications^a^
1	F	Right	Vehicle accident	II	No	Anatomical	NO
2	M	Right	High fall injury	III	Subtalar joint and talonavicular joint	Anatomical	NO
3	M	Right	High fall injury	II	No	Anatomical	NO
4	M	Left	Crushing	II	No	Anatomical	NO
5	M	Right	High fall injury	II	No	Anatomical	NO
6	F	Left	High fall injury	II	No	Anatomical	NO
		Right	High fall injury	III	No	Anatomical	NO
7	F	Right	High fall injury	II	No	Anatomical	NO
8	M	Left	High fall injury	II	No	Nearly anatomical	Osteoarthritis
9	M	Right	High fall injury	II	No	Anatomical	NO
10	M	Left	Twisting injury	II	Ankle joint and subtalar joint	Anatomical	NO
11	F	Right	Twisting injury	II	No	Anatomical	NO
12	F	Right	Twisting injury	II	No	Anatomical	NO
13	M	Right	High fall injury	II	No	Anatomical	NO
14	M	Right	High fall injury	II	Subtalar joint and talonavicular joint	Anatomical	Osteoarthritis
15	M	Left	Vehicle accident	II	No	Anatomical	NO

*F *Female, *M* Male

^a^There were no complications of skin infection, skin necrosis, osteomyelitis, osteonecrosis, or non-union

### Complications and clinical outcomes

No skin infection, osteomyelitis, or skin necrosis was observed in any patient. Osteonecrosis, bony nonunion, or delayed union was not found during the follow-up.

At the final follow-up, 2 out of the 15 patients (13.3%) had peri-talar osteoarthritis. The ankle–hindfoot pain was absent in 11 patients (12 ankles) and mild in 4 patients. The wound-scar score ranged from 0 to 3 points (mean, 0.63 points). The ROM of the ankle joint was normal or mild restriction in 10 ankles, and moderate restriction in 6 ankles. The sub-talar joint mobility was moderate restriction in 9 ankles and severe restriction in 7 ankles. These results indicated that the injured foot demonstrated a loss of ROM for the ankle and sub-talar joint.

**Fig. 4:**
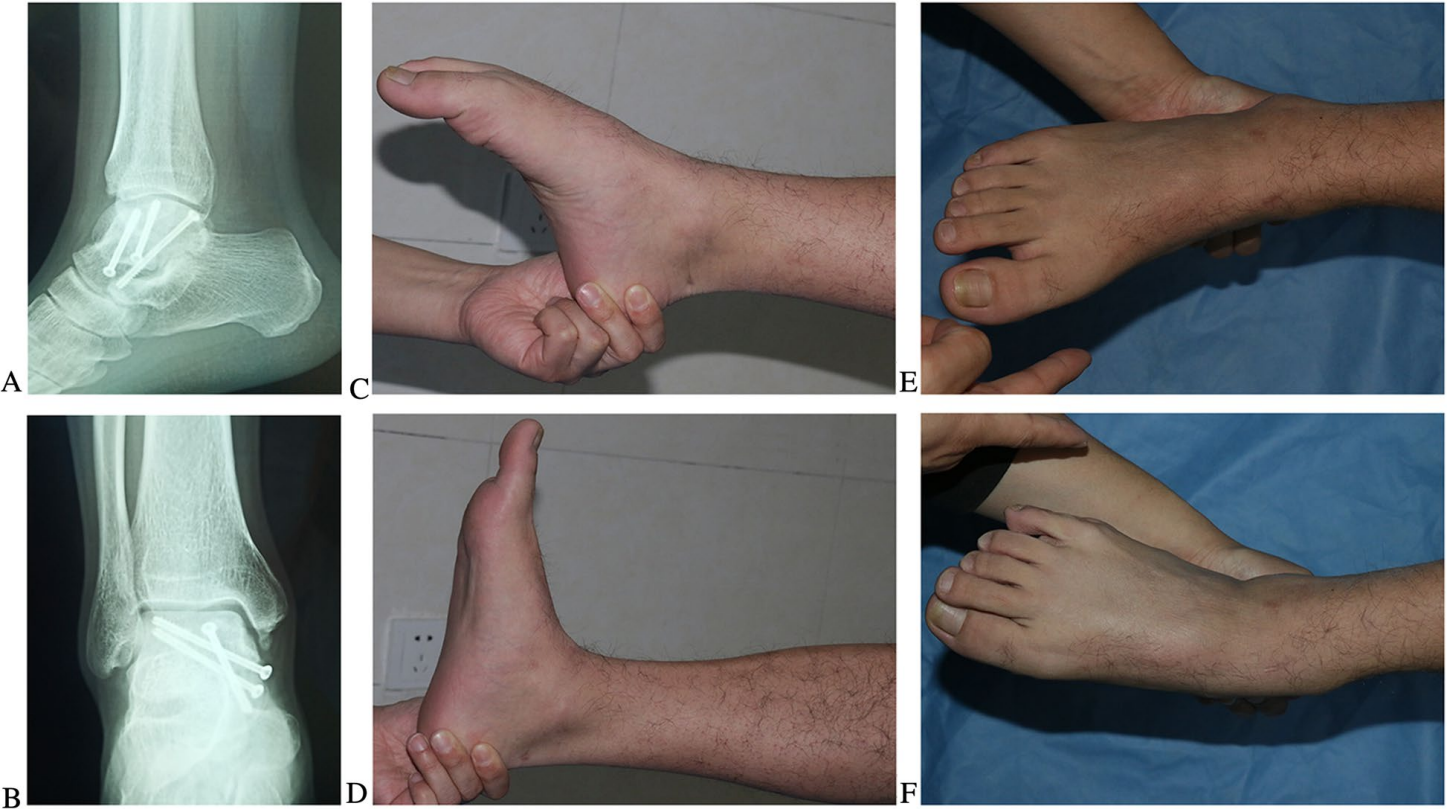
Results of the arthroscopically assisted percutaneous talar osteosynthesis of a talar body fracture (Sneppen II) in the right ankle of a young male patient 35 months post-operation

Based on the AOFAS ankle–hindfoot scores, the functional results at the final follow-up were excellent in 7 ankles and good in 9 ankles. The mean AOFAS ankle–hindfoot and MOxFQ scores of the patients were 85.7 (range, 79–93) and 6.48 (range, 0–12.5) points, respectively. Accordingly, the patients showed satisfactory results (Fig. 4).

**Fig. 5:**
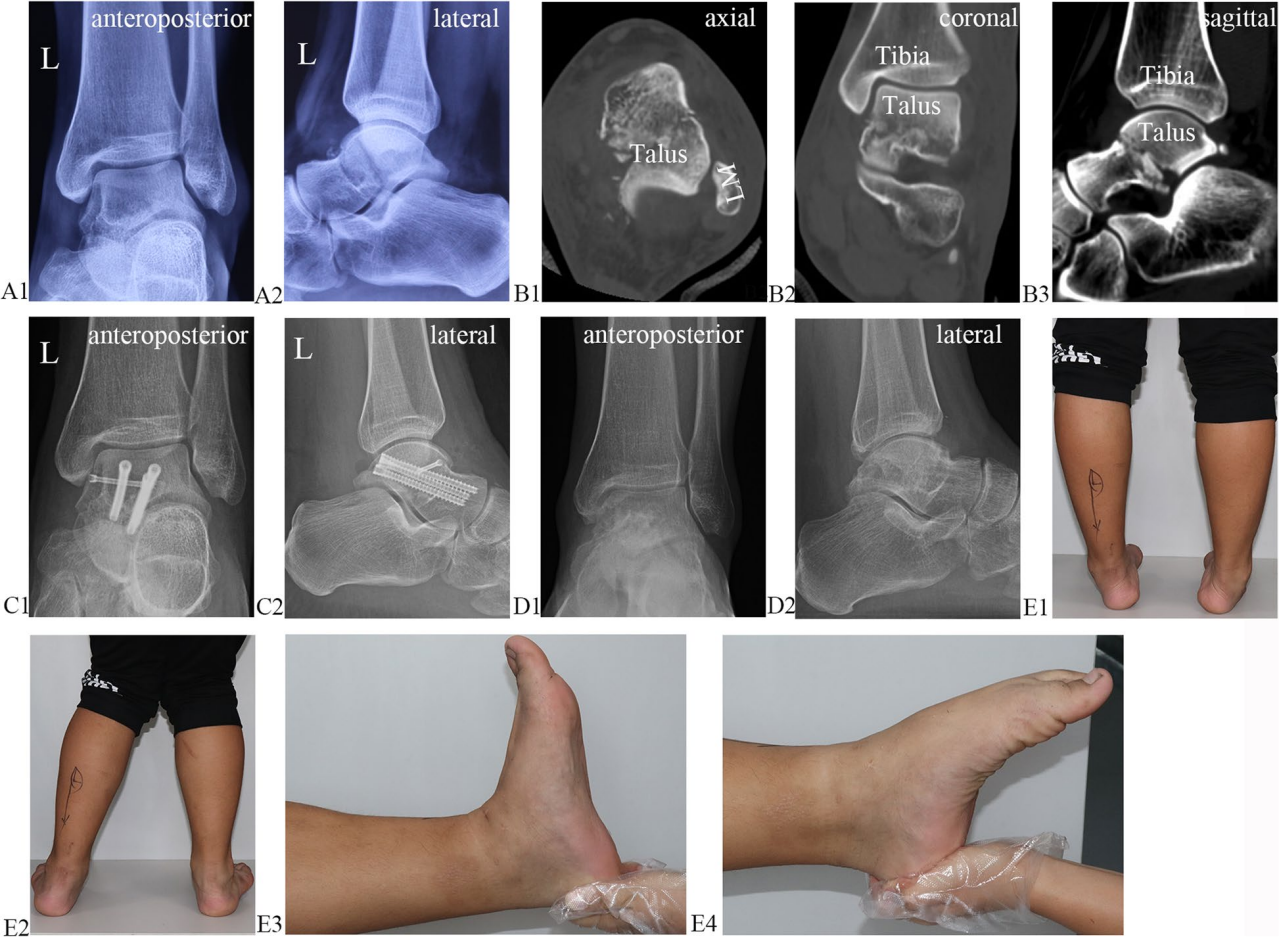
Preoperative X-ray radiography (**A1** and **A2**) and CT (**B1**–**B3**) results. X-ray radiography results one week (**C1**–**C2**) and two years (**D1**–**D2**) after the operation. Functional results two years after the operation (**E1**–**E4**)

**Fig. 6:**
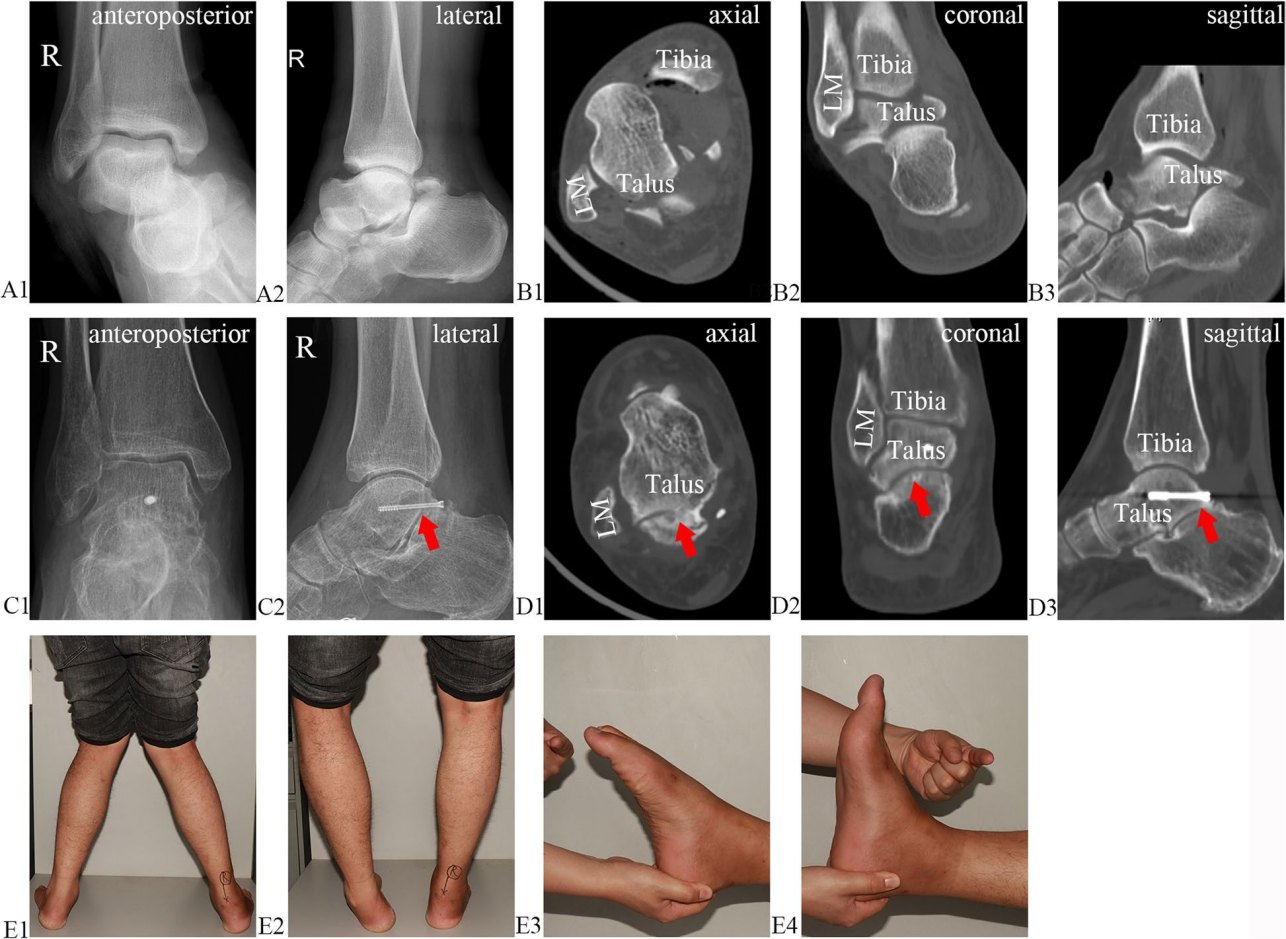
X-ray radiography and CT results before (**A1–2** and **B1–B3**, respectively) and 18 months after (**C1–2** and **D1–D3**, respectively) the operation. LM: lateral malleolus. Red arrow: sub-talar osteoarthritis. Functional results 18 months after the operation (**E1–E4**)

### Case reports

#### Case 1 (patient 15)

A 22-year-old man sustained a car accident, resulting in fractures on the left talar body, which were identified via X-ray radiography (Fig. 5A1 and A2) and CT (Fig. 5B1–B3). The patient underwent arthroscopically assisted percutaneous osteosynthesis. A radiographic examination was performed one week after the operation (Fig. 5C1 and C2), and the hollow screws were removed one year after the operation. No peri-talar osteoarthritis was found on the radiograph two years after the operation (Fig. 5D1 and D2). The patient showed a satisfactory functional result (Fig. 5E1–E4).

#### Case 2 (patient 14)

A 36-year-old male patient suffered from fractures on the right talar body, and sub-talar and talonavicular joint dislocation (Fig. 6A1–B3) due to a high fall. For the treatment, arthroscopically assisted percutaneous osteosynthesis was performed. After 18 months, the patient presented with sub-talar osteoarthritis (Fig. 6C1–D3), and the functional recovery of the ankle and sub-talar joints is shown in Fig. 6E1–E4. The mobility scores of the ankle and sub-talar joints were 2 and 1 points, respectively. The AOFAS ankle–hind-foot and MOXFQ scores were 79 and 12.5 points, respectively.

## Discussion

In this study, our results demonstrated that arthroscopically assisted percutaneous talar osteosynthesis is a reliable and feasible procedure and provides good clinical outcomes with a low risk of complications, although to a level still less than that on the healthy foot.

Unlike the conventional ORIF [6, 8], the arthroscopically assisted percutaneous talar osteosynthesis technique can avoid the requirement of medial or lateral malleolar osteotomy for exposure. Arthroscopically assisted percutaneous talar osteosynthesis can effectively protect the fragile soft tissue surrounding the injury and lower the risk of damage to the anatomical structures and vasculature surrounding the talus. Moreover, the associated wounds are often scar-free. In contrast to the ORIF [6, 8] and the technique of Abdelgaid et al. [10], arthroscopy can be used to easily identify and evaluate intra-articular loose bodies, cartilage injuries, and trans-chondral defects that may not be detectable on X-ray radiographs. Furthermore, it can aid the surgeons to visualize and manipulate joint structures, control the reduction, position the osteosynthesis devices, and perform a stable fixation without arthrotomy, whereby any protrusion or dislocation of the implants is avoided. Moreover, both the technique of Abdelgaid et al. [10] and ours have the advantage of early minimally invasive fracture reduction and fixation, and rapid rehabilitation. However, the drawbacks of our technique are the long learning curve and operative time. Nevertheless, talar body fractures treated with ORIF usually present with a much higher incidence of postoperative complications, such as infection, skin necrosis, peri-talar osteoarthritis, and avascular necrosis (Table 2) [5, 9, 13, 19–21]. Additionally, previous studies have concluded that the severity and incidence of these complications are associated with the level of the injury to the soft tissue surrounding the ankle, the extent of intrinsic vascular injury following the talar fractures, the extent of fracture displacement, the absence or presence of joint dislocation, and the adequacy of fracture reduction, but not related to the operative time [9, 22, 23]. As shown in Table 2, peri-talar osteoarthritis and avascular osteonecrosis were the most common complications after the assisted percutaneous osteosynthesis of the talar body fractures. However, no bony nonunion or delayed union was found in any of the patients during the follow-up period possibly because (1) the procedure causes little damage to the tissues surrounding the talus, thus effectively protecting the fragile soft tissue around the injury and lowering the risk of damage to the vasculature around the talus; (2) the anatomical reduction rate was very high; and (3) the state of fracture union and the presence of osteonecrosis or osteoarthritis may be underestimated in the radiographic examination.

**Table 2:** AOFAS scores and complication rates of the talar body fractures treated with conventional open reduction-internal fixation

Authors	Year	Age (Year) (mean, range)	Number of patients (talus)	Number of talar body fracture	Infection	Skin necrosis	Peri-talar osteoarthritis	Avascular necrosis	AOFAS Ankle-Hindfoot Score (mean, range)
Elgafy [19]	2000	32 (14–74)	58 (60)	11	18.2%	N/A	Subtalar arthritis: 90.9%, Ankle arthritis: 90.9%	27.3%	58 (10–90)
Vallier [5]	2003	34 (15–74)	56 (57)	57	7.1%	1.8%	Subtalar arthritis: 34.6%, Ankle arthritis: 65.4%	38.5%	NR
Ebraheim [20]	2008	31 (21–68)	19 (19)	19	15.8%	5.3%	Subtalar arthritis: 31.6%, Ankle arthritis: 57.9%	36.8%	68.6 (44–94)
Gomes de Sousa [21]	2009	38.5 (14–63)	19 (19)	5	N/A	N/A	Subtalar arthritis: 50.0%, Ankle arthritis: 40%	45.5%	72 (19–100)
Ohl [13]	2011	38.8 (17–76)	20 (20)	10	10%	0%	Subtalar arthritis: 87%, Ankle arthritis: 76%	20.0%	67 (45–88)
Biz [9]	2019	38.3 (18–81)	31(33)	18 (19)	0%	5.3%	Subtalar arthritis: 36.8%, Ankle arthritis: 31.6%	15.8%	81 (55–97)

*NR* No report

Talar body fractures can influence the normal joint motion of the ankle and lead to a poor prognosis in the long term [24]. As previously reported [5, 9, 13, 19–21], patients with ORIF-treated talar body fractures often have low AOFAS scores. The AOFAS ankle–hindfoot scores of these patients range from 58 to 81 points, whereas the mean score of the patients in our group was 85.7 points (range, 79–93 points) (Table 2). However, compared with arthroscopically assisted percutaneous talar osteosynthesis, we found that the biggest disadvantage of ORIF is the difficulty in minimizing the damage to the healthy tissue surrounding the talus, for maintaining the talus blood supply and providing a large enough field for fracture reduction and internal fixation. Furthermore, conventional surgical approaches usually cannot fully expose the talus body, thus necessitating medial or lateral malleolar osteotomy and risking the integrity of the remaining blood supply, not to mention the possibility of talus nonunion and malunion.

Currently, ankle arthroscopy is becoming increasingly prevailing in the diagnosis and treatment of ankle diseases and fractures [25, 26]. An intra-articular displacement of > 2 mm can change the biomechanics of the ankle joint [27]. In our study, 15 of the 16 talus fractures achieved anatomical reduction, and 1 fracture achieved nearly anatomical reduction. In our experience, the more severe the talar body fracture, the more difficult it is to achieve anatomical reduction under arthroscopy. The indications of arthroscopically assisted percutaneous talar osteosynthesis are as follows: (a) talar body fractures of Sneppen I–IV, (b) articular displacement > 2 mm, (c) soft-tissue swelling with fracture blisters, and (d) multiple (ankle, sub-tala, or talonavicular) joint dislocations permissive to closed reduction.

This retrospective study has some limitations. First, a combined joint dislocation is highly coupled with the possibility of vascular injury around the talus, which can affect the incidence of osteoarthritis or avascular necrosis of the talus after fracture reduction. Unfortunately, we could not determine the level of vascular injury around the talus after joint reduction. Second, the state of fracture union and the presence of osteonecrosis or osteoarthritis may be underestimated in the radiographic examination, and the absence of a long-term follow-up via CT or MRI is also another limitation of this study. Finally, the follow-up time was relatively short, and the sample size was small. Thus, our conclusions require confirmation, and the pros and cons of the traditional ORIF and arthroscopically assisted percutaneous talar osteosynthesis should be compared in a multicenter study with a large sample size and long follow-up period in the future.

## Conclusions

Arthroscopically assisted percutaneous talar osteosynthesis is a reliable and feasible technique and provides good clinical outcomes and a low risk of complications, although to a level still less than that on the healthy foot.

## Data Availability

All data generated or analysed during this study are included in this published article and its supplementary information files.

## Abbreviations

AOFAS: American Orthopaedic Foot and Ankle Society
ORIF: Open reduction-internal fixation
CT: Computed tomography
mVSS: modified vancouver scar scale
MOxFQ: Manchester-Oxford foot questionnaire
